# Scleral flaps, pars plana vitrectomy and gore-tex sutured posterior chamber intraocular lens placement: a case series and review of literature

**DOI:** 10.3389/fopht.2023.1147881

**Published:** 2023-07-26

**Authors:** Pasquale Napolitano, Mariaelena Filippelli, Marianna Carosielli, Ciro Costagliola, Roberto Dell’Omo

**Affiliations:** Department of Medicine and Health Sciences “V. Tiberio”, University of Molise, Campobasso, Italy

**Keywords:** scleral fixation IOL, scleral flaps, pars plana vitrectomy, gore-tex suture, intraocular lens

## Abstract

**Introduction:**

Cataract surgery is one of the most common surgical procedures performed worldwide. Intraocular lens (IOL) implants are placed routinely in the capsular bag after successful cataract extraction. However, in the absence of adequate capsular support, IOL may be placed in the anterior chamber, fixated to the iris or fixated to the sclera. The purpose of this study is to report the clinical outcomes and safety profile of a trans-scleral sutured intraocular lens (IOL) technique using scleral flaps, vitrectomy, and Gore-Tex suture to place posterior chamber IOL.

**Methods:**

Retrospective, interventional case series of eyes undergoing scleral fixation of an IOL using Gore-Tex suture with concurrent vitrectomy. Ocular examination with the logarithm of the minimum angle of resolution visual acuity (logMAR BCVA), tonometry, and slit-lamp biomicroscopy was performed on all patients at 1, 3, 6, and 12 months after the operation. All post-operative complications were recorded.

**Results:**

Twenty-five eyes of 25 patients were included. Mean logMAR BCVA improved from 0.43 ± 0.36 (20\40 Snellen equivalent) preoperatively to 0.13 ± 0.18 (20\25 Snellen equivalent) postoperatively at 12 months (p<0.01). Indications included surgical aphakia (16) and dislocated lens implant (9). No cases of IOL opacification, suprachoroidal haemorrhage, post-operative endophthalmitis, IOL dislocation, Gore-Tex exposure, or retinal detachment were observed during the follow-up period.

**Conclusion:**

Ab externo scleral fixation of IOLs with Gore-Tex suture plus scleral flap is well tolerated and associated with a very low rate of suture exposition. Moreover, our study confirms excellent refractive outcomes after surgery.

## Introduction

Cataract surgery is one of the most common surgical procedures performed worldwide, with more than 9.5 million surgeries performed yearly (1). Intraocular lens (IOL) implants are placed routinely in the capsular bag after successful cataract extraction. However, in the absence of adequate capsular support, IOL may be placed in the anterior chamber, fixated to the iris or fixated to the sclera (2, 3). Although a recent assessment by the American Academy of Ophthalmology (4) found no differences in clinical and visual outcomes of any one method of secondary implantation (5), the main advantage of scleral fixation of IOL over the other techniques is the positioning of the IOL away from anterior chamber structures. In clinical scenarios where posterior segment disease must also be addressed, such as retained lens material, dislocated IOL, and vitreous prolapse, scleral fixation of IOL is performed in combination with pars plana vitrectomy (PPV) (6, 7).

For many years, prolene (polypropylene; Ethicon, Somerville, New Jersey, USA) suture has been used for scleral-fixated IOL. More recently, scleral fixation of a posterior chamber IOL using Gore-Tex (W.L. Gore & Associates, Newark, DE) suture, a nonabsorbable, polytetrafluoroethylene monofilament suture with greater tensile strength and a presumed decreased risk of suture breakage in comparison to prolene, has been described with positive outcomes, i.e. no suture-related complications, and significant improvement in best-corrected visual acuity (8, 9).

However, routinely using Gore-Tex suture to place scleral-fixated IOL since 2017, we found that erosion of the conjunctiva overlying the suture may develop over time in several cases. Another limitation of the Gore-Tex technique is that rotation of the knot into sclerotomy at the end of the surgical procedure may result in decentration of the lens. In order to address these issues, we constructed partial-thickness scleral flaps to cover the Gore-Tex suture and knot after accurate IOL centration.

This study aims to report the clinical outcomes and safety profile of a trans-scleral sutured IOL technique using scleral flaps, vitrectomy, and Gore-Tex suture.

## Methods

This retrospective, interventional case series was conducted following regulations set forth by the Health Insurance Portability and Accountability Act. The Institutional Review Board of the University of Molise approved this study. All research adhered to the tenets of the Declaration of Helsinki. As this was a retrospective chart review, the Institutional Review Board waived the need to obtain informed consent from patients.

All patients were treated at the Department of Ophthalmology, University of Molise, Campobasso, Italy.

The electronic medical records for all patients who underwent scleral fixation of an IOL using Gore-Tex suture with concurrent vitrectomy were reviewed from January 1, 2018, through November 30, 2021.

The indication for PPV and secondary IOL placement with Gore-Tex scleral fixation was dislocated IOL and aphakia with poor capsular support.

The following data were collected: age, gender, the preoperative and post-operative logarithm of the minimum angle of resolution (logMAR), best-corrected visual acuity (BCVA) measured on the Early Treatment Diabetic Retinopathy Study (ETDRS) charts at 4 m preoperative and post-operative, tonometry for intraocular pressure (IOP), keratometry values, axial length, macular study with Spectral-Domain optical coherence tomography (OCT), and intraoperative and post-operative outcomes. Patients with retinal detachment, epiretinal membrane, cystoid macular oedema, retinal vein occlusion, age-related macular degeneration, or diabetic retinopathy were excluded.

Conventional biometry was used for IOL calculations with Lenstar Biometry (Haag Streit, Koeniz, Switzerland) The expected post-operative spherical equivalent (SE) was calculated using the Sanders-Retzlaff-Kraff theoretical (SRK/T) formula assuming in-the-bag IOL location. Each patient underwent clinical examination 1, 3, 6, and 12 months after surgery.

**Figure 1:**
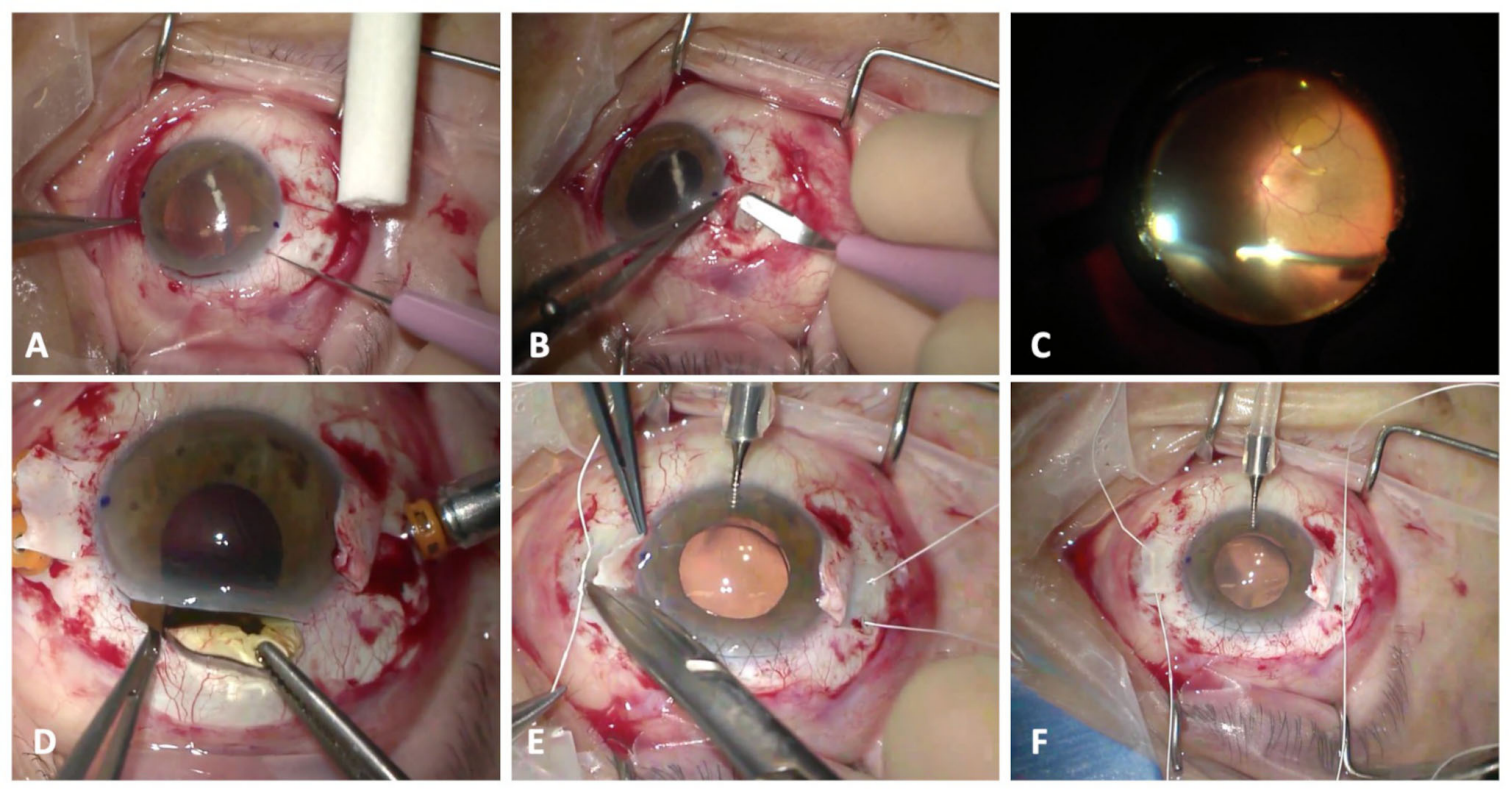
**(A, B)**. Nasal and temporal scleral flap of 4 mm x 4 mm was practiced. **(C)**. A standard pars plana vitrectomy is performed **(D–F)**. The IOL was introduced into the anterior chamber and the sutures were tied using a a 3-1-1 or slip-knot technique to center the IOL. The suture knots were trimmed and rotated into a sclerotomy site.

Twenty-five patients who underwent scleral fixation of a posterior chamber IOL and for whom preoperative and post-operative refractive data were available were included in the final analysis. The implanted IOL was, in all cases, a single-piece hydrophobic lens. A single surgeon (RdO) performed all the surgical procedures using the same technique (Online Resource 1). In brief, after corneal demarcation at 0° and 180°, a 360° peritomy was performed. Nasal and temporal scleral flaps of 4 × 4 mm were sculpted (**Figures 1A, B**) with an implant of 4 self-retaining 23-gauge valved cannulas about 3 mm apart at each corner of the scleral flap and at 3.5 mm from the limbus. Then, a thorough pars plana vitrectomy with accurate shaving of the vitreous base under scleral depression (**Figure 1C**) was performed using the Constellation platform (Alcon, Fort Worth, TX, USA). A perilimbal 2.5 mm or 7 mm wide sclero-corneal was made in the superior limbus in case of aphakia or dislocated IOL, respectively. The CV-8 Gore-Tex suture was cut into equal halves and laced through each pair of adjacent eyelets of a hybrid copolymer IOL (ISP60Z i-stream h, MD tech, Casoria, Italy). In a hand-to-hand technique, each end of the Gore-Tex suture was passed into the anterior chamber and externalized from each corresponding trocar using serrated forceps (Alcon, Grieshaber Revolution DSP, Fort Worth, TX, USA). The IOL was then introduced into the anterior chamber, and the sutures were tied using a 3-1-1 or slip-knot technique to centre the IOL. The suture knots were trimmed and rotated into a sclerotomy site, provided that this manoeuvre did not cause decentration. (**Figures 1D–F**). In those cases where the rotation caused a decentration of the IOL, the knot was left unburied and simply covered with the scleral flap. The corneal incision was sutured with a 10.0 nylon running suture, whereas the scleral flaps and the conjunctiva were sutured with 7.0 vycril sutures. Corneal sutures were removed 3 months after surgery.

All post-operative complications were recorded. Hypotony was defined as a new onset of an IOP of ≤ 5 mm Hg at any post-operative visit, corneal oedema was defined as new-onset post-operative oedema that persisted >1 month and macular oedema as new-onset post-operative oedema that was confirmed with optical coherence tomography (OCT).

For statistical analysis, Snellen visual acuities were converted to logarithm of the minimum angle of resolution (logMAR) equivalents for statistical analysis. Descriptive statistics are summarized as the mean ± standard deviation. Axial length measurements were obtained using either optical biometry (Lenstar Biometry, Haag-Streit) or contact ultrasonography (Quantel Medical by Lumibird Medical, Cournon d’Auvergne, France). Automated keratometry readings were obtained using the Lenstar Biometry. A-constants for lenses were used as suggested by the manufacturer. The IOL power implanted in each patient was used to calculate predicted spherical outcomes for each formula assessed. All calculations were carried out assuming in-the-bag IOL positioning. The SE was used for the analysis of post-operative refractive data. In assessing refractive outcomes, each IOL formula calculated the prediction error as the post-operative SE refraction minus the predicted refraction. Thus, a negative prediction error indicates a more myopic final refraction than the IOL formula calculation predicted. Mean absolute prediction errors were calculated as the absolute value of the difference between the SE and predicted refractions. Overall refractive outcomes were also compared with the patient’s target refraction. Visual outcomes were analyzed using a one-way ANOVA test analysis performed using GraphPad Prism (GraphPad Software Inc., La Jolla, CA). A p-value of 0.05 was considered statistically significant.

## Results

A total of 25 eyes of 25 patients (13 right and 12 left eyes) were included. Of the 25 patients, 15 (60%) were men. The mean age at the time of surgery was 68.8 ± 10.14 years (range, 52–82). Baseline characteristics of patients are shown in **Table 1**. Sixteen patients underwent secondary Gore-Tex implantation of IOL because of surgical aphakia and nine because of IOL subluxated in the vitreous cavity.

**Table 1:** Patient characteristics.

Characteristics		Mean (± SD)
No. of eyes		25
Age (years)		68.8 (±10.14)
Gender (male/female)		15/10
Diagnosis (no. of eyes)		
	Dislocated PC IOL	9
	Surgical aphakia	16

**Figure 2:**
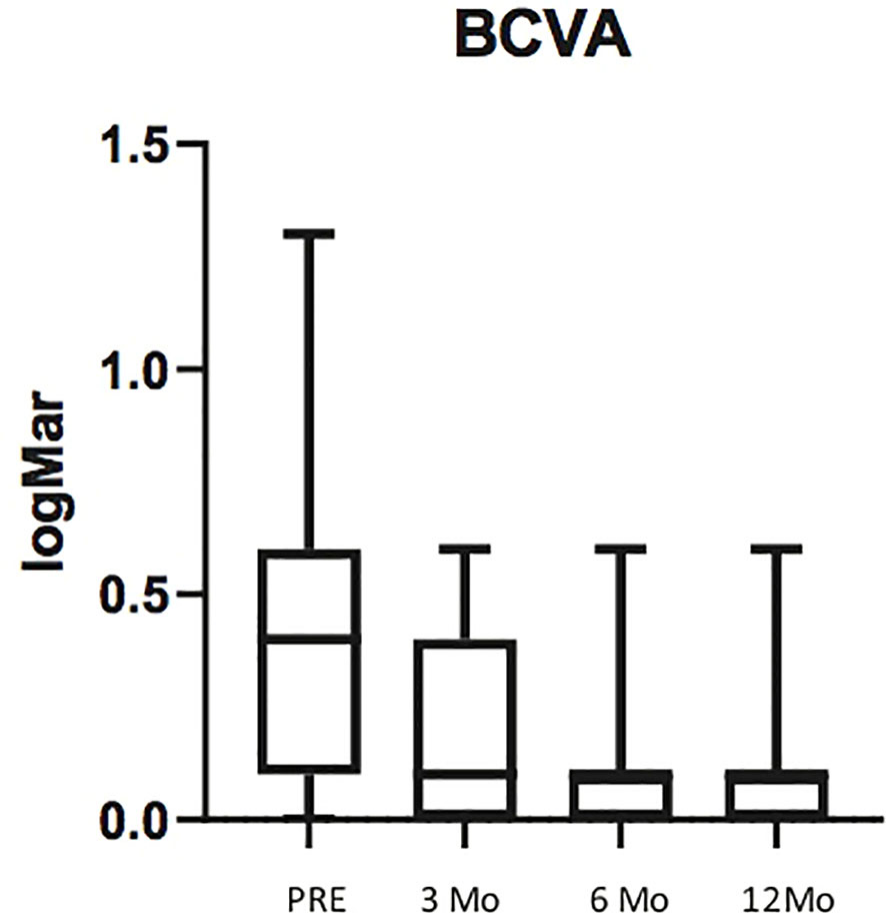
Box plots illustrating preoperative Best Corrected Visual Avcuity (BCVA) and BCVA at 3, 6 and 12 months post surgery. The horizontal lines within each box represent the median for each group, the ends of the boxes are the upper and lower quartiles, and the whiskers represent minimum and maximum values.

Mean preoperative logMAR BCVA was 0.43 ± 0.36 (20/40 Snellen equivalent), with 13 eyes (52%) having ≥ 20/40 and 23 eyes (92%) with ≥ 20/200 Snellen equivalent BCVA. Mean logMAR BCVA improved at 0.19 ± 0.23 (20/32 Snellen equivalent) (p<0.06) 3 month after operation, at 0.16 ± 0.22 (20/32 Snellen equivalent) 6 months after operation (p<0.03), and at 0.13 ± 0.18 (20\25 Snellen equivalent) 12 months after operation (p<0.01) (**Figure 2**). The mean change in logMAR BCVA was 0.29 ± 0.18.

The mean attempted SE was -0.29 ± 0.92D, while the mean achieved SE at 12months was -0.97 ± 0.83 D. Average predictive error (post-operative spherical equivalent refraction minus target refraction) was -0.68 ± 0.09 D, indicating a trend to ward slightly myopic outcomes than targeted.

**Figure 3:**
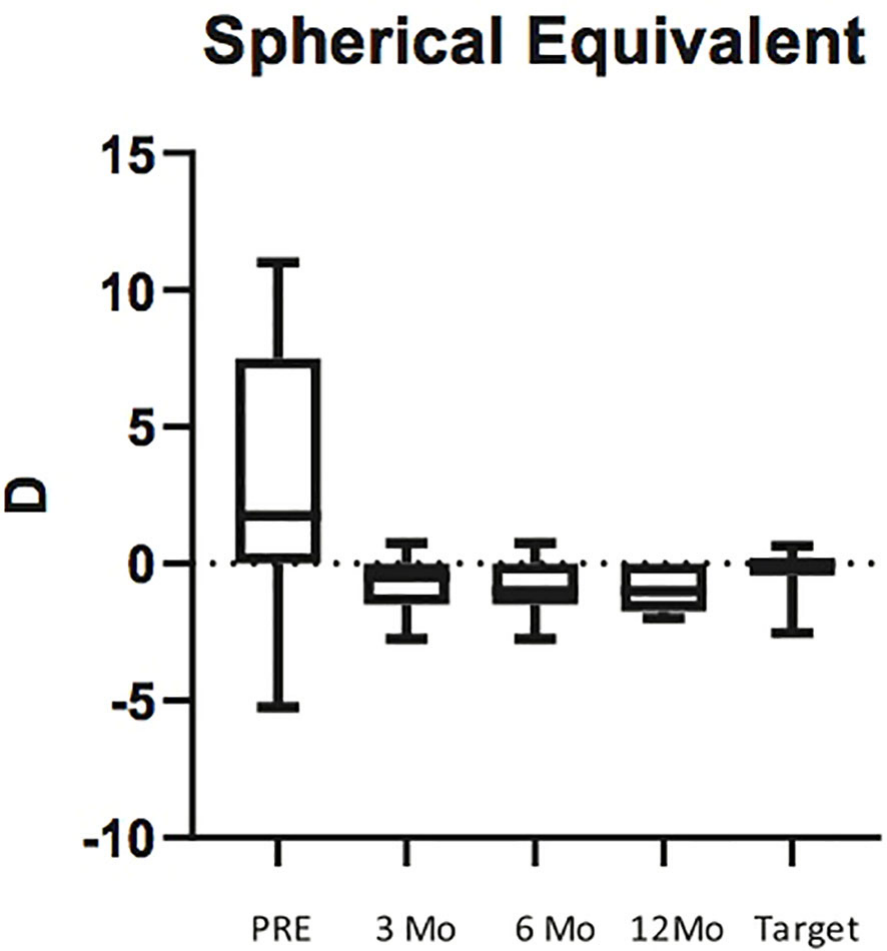
Box plots illustrating preoperative Spherical Equivalent (SE) and SE at 3, 6 and 12 months post surgery. The horizontal lines within each box represent the median for each group, the ends of the boxes are the upper and lower quartiles, and the whiskers represent minimum and maximum values.

There was no significant difference between SE calculated at 3 and 6 months (p<0.99) and between SE calculated at 6 and 12 months (p>0.99) (**Figure 3**).

**Figure 4:**
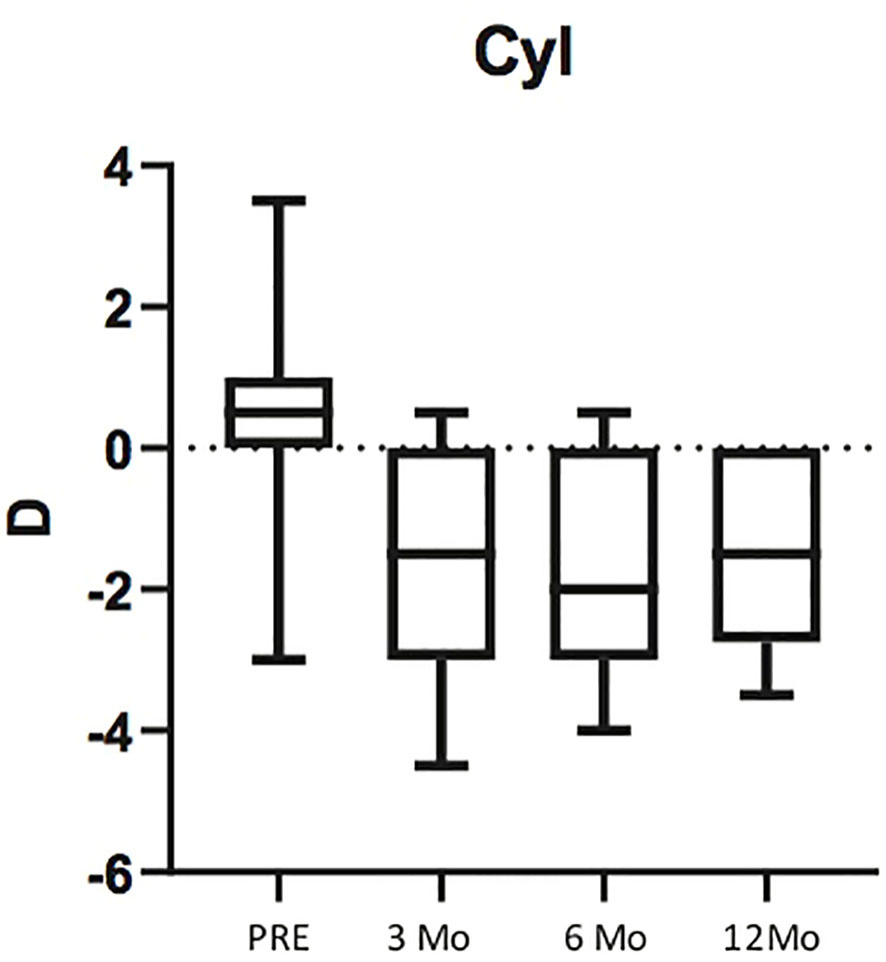
Box plots illustrating preoperative Astigmatism and Astigmatism at 3, 6 and 12 months post surgery. The horizontal lines within each box represent the median for each group, the ends of the boxes are the upper and lower quartiles, and the whiskers represent minimum and maximum values.

Astigmatism varied significantly between preoperative and 3 months after surgery (p<0.003), while there was no significant difference between astigmatism values measured at 3 and 6 months (p<0.99) and between astigmatism values measured at 6 and 12 months (p<0.98) (**Figure 4**; **Table 2**).

**Table 2:** Surgical outcomes.

	logMAR BCVA	p value	SE (D)	p value	Astigmtism (D)	p value
**Preoperative (mean ± SD)**	0.43 ± 0.36		2.80 ± 4.85		0.37 ± 1.51	
**3 months post surgery (mean ± SD)**	0.19 ± 0.23	<0.06	-0.70 ± 0.88	**<0.002** ^✽^	-1.63 ± 1.72	**<0.003**☩
**6 months post surgery (mean ± SD)**	0.16 ± 0.22	**<0.03** ^＊^	-0.95 ± 0.99	<0.99	-1.70 ± 1.48	<0.99
**12 months post surgery (mean ± SD)**	0.13 ± 0.18	**<0.01**☥	-0.97 ± 0.83	>0.99	-1.50 ± 1.31	<0.98

BCVA, Best Corrected Visual Acuity; SE, Spherical equivalent; D, Diopter.

The bold values indicate significant results.

^*^Significant difference (p<0.03) between mean values in preoperative BCVA and BCVA at 6 months post surgery comparison.

☥ Significant difference (p<0.01) between mean values in preoperative BCVA and BCVA at 12 months post surgery comparison.

^✽^Significant difference (p<0.02) between mean values in preoperative SE and SE at 3 months post surgery comparison.

☩ Significant difference (p<0.03) between mean values in preoperative Astigmatism and Astigmatism at 3 months post surgery comparison.

We did not observe cases of IOL opacification, suprachoroidal haemorrhage, post-operative endophthalmitis, IOL dislocation, Gore-Tex exposure, or retinal detachment during the 6-month follow-up period.

## Discussion

Many techniques for secondary IOL implant in an eye with inadequate capsular support exist. To our knowledge, there is no evidence of the superiority of anyone in clinical or visual outcomes (5, 10).

We performed a systematic review of the literature using search keyword gore-tex fixated IOL and scleral fixation IOL.

In their study of 2019, Su and coll analyzed fifty-five eyes from 53 patients who underwent PPV with a Gore-Tex sutured IOL, studying the refractive outcomes. They found a more myopic outcome when the IOL was fixated 2 mm from the limbus compared with 3 mm from the limbus. Furtheremore, they found no significant difference between IOL models (11).

Huang et al. in their retrospective, interventional, consecutive case series compared the classic technique required a relatively large corneal wound or scleral tunnel incision that ranges from 3.5 to 7 mm depending on the IOL model and their novel technique with 2.3mm corneal incision. They concluded that the modified small-incision technique for implantation of scleral-fixated IOLs using Gore-Tex sutures was well tolerated in all patients, with favorable postoperative visual outcomes and minimal surgically induced astigmatism (SIA) and IOL-induced astigmatism (12).

In studies using polypropylene sutures for IOL fixation, the most prominent postoperative complication was suture erosion and breakage which was largely avoided in the use of Gore-Tex sutures (13). Few complications related to Gore-Tex sutures used in scleral fixation of IOLs have been reported. Moreover, none have reported suture breakage postoperation, although the follow-up duration was too limited in most of the studies to make a conclusion.

In a recent study on Journal of VitreoRetinal Diseases authors analyzed the complication related to different kind of suture used for IOL fixation. Prolene (Ethicon, Inc) was proven to be well tolerated for intra-ocular use. Nevertheless, its tendency to break down over time has led many to reexamine its benefits (14). One report found that 10-0 Prolene breakage occurred in 27% of eyes that underwent scleral-sutured IOL, and approximately half (49%) of eyes required additional procedures to resolve postoperative complications (15). For this reason, Gore-Tex has gained interest for replacement of Prolene in scleral-sutured IOLs because of its documented success in nonophthalmic procedures (16). An original article published on September 2022 compared sutureless flanged fixation and 4-point Gore-Tex fixation for scleral-fixated intraocular lenses. Authors concluded that both techniques offer excellent visual rehabilitation with minimal complication rates. In their study, the Gore-Tex sutured group had better postoperative visual acuity, IOL centration, and stability (17).

On the opposite side, in a recent paper, Canabrava et al. assessed the long-term (5-year) results and complications of the double-flanged polypropylene technique in patients with capsular tension segment fixation, non-foldable IOL scleral fixation, and foldable IOL scleral fixation. Placing before that principal complications are long-term polypropylene resistance, conjunctival erosion, conjunctival inflammation, flange exposure, internalization, endophthalmitis, retinal detachment, and cystoid macular edema. They concluded that the double-flanged technique was proven to be stable when the correct technical procedure was followed. However, complications can be observed, especially with short scleral tunnels and in eyes where the flanges were not buried inside the sclera (18).

We reported our experience regarding secondary IOL implant using Gore-Tex sutures and scleral flaps.

Complications of scleral-fixated IOLs most often include IOL tilt and suture erosion or breakage over time (19, 20). Our technique allows us to overcome the problem of degradation of the prolene suture and the subsequent dislocation of the IOL using Gore-Tex, a nonabsorbable monofilament suture with great resistance to rupture. Gore-Tex sutures allow for a very stable, 4-point lens fixation reducing the risk of inflammation secondary to contact of the IOL with the iris.

The innovation of our technique, compared to other techniques of ab externo scleral fixations of IOLs with Gore-Tex suture, consists of the use of partial-thickness scleral flaps. We have noticed that the rotation of the knot of Gore-Tex suture into sclerotomy at the end of the surgical procedure may result in decentration of the lens. However, rotation of the knot and consequent IOL dislocation may be avoided by sculpting scleral flaps that cover the knot. Furthermore, covering the Gore-Tex knot under scleral flaps also reduces the chance of suture exposition. Finally, the scleral flaps may better seal the sclerotomies reducing the risk of post-operative hypotony, one of the most common post-operative complications following secondary, scleral-fixated IOL (21, 22).

Many studies assessing postoperative refraction in eyes with scleral-fixated IOLs, have reported a myopic shift from biometry target (19, 20, 23, 24). Our study confirms this data. We used the SRK/T formula with optical biometry to calculate IOL power. This predictive lens formula was designed assuming that an IOL would be placed in the capsular bag while scleral-fixated lenses are placed in the absence of capsular support. The inaccuracy in calculating the power of the IOL of this formula may depend on errors in measuring the axial length and the effective lens position, which is variable in patients with IOL subluxation. Nevertheless, in our study, the myopic shift was very low, suggesting that our technique provides good refractive results while keeping the standard distance of trocars position from the limbus (i.e., 3.5 mm) used for routine pars plana vitrectomy.

In the current study, BCVA progressively increased during follow-up and the improvement was, statistically significant at 6 months after surgery. Astigmatism significantly increased after surgery in the whole group, likely due to the corneal suture in the eyes where a 7 mm corneal incision was made to extract the luxated IOL. In fact, no significant post-operative astigmatism was observed in the sixteen aphakic eyes in which a 2.5 mm corneal tunnel was created. Nevertheless, astigmatism in the 7 mm corneal incision eyes stabilized between the first and third months after surgery, and the SE did not vary significantly after the suture removal.

A strength of our study is that all the procedures were performed by the same surgeon, reducing the variability related to the experience of different operators. This study has also several limitations, many of which are inherent to its retrospective nature and the small sample of patients.

In conclusion, our experience with this technique has been positive. Indeed, it reduces the risk of the most common complications related to this kind of surgery and obtains excellent refractive results. However, long-term evaluation of outcomes and complications will be necessary.

Prospective, comparative trials would be necessary to determine the superiority of this technique compared with alternative IOL implantation strategies.

## Data availability statement

The original contributions presented in the study are included in the article/**Supplementary Material**. Further inquiries can be directed to the corresponding author.

## Ethics statement

The studies involving human participants were reviewed and approved by Institutional Review Board of the University of Molise. The patients/participants provided their written informed consent to participate in this study.

## Author contributions

Conceptualization: RD’O. Methodology: PN, MF, RD’O. Formal analysis and investigation: MF, R’DO. Writing - original draft preparation: PN, MC, R’DO. Writing - review and editing: PN, CC, R’DO. Resources: MC, CC. Supervision: CC, R’DO. All authors contributed to the article and approved the submitted version.

## References

[1] Micieli JA Arshinoff BSc and SA. Cataract surgery. Can Med Assoc J (2011) 183(14):2011. doi: 10.1503/cmaj.110549 PMC3185079 21825045

[2] Por YM Lavin MJ. Techniques of intraocular lens suspension in the absence of capsular/zonular support. Surv Ophthalmol (2005) 50(5):429–62. doi: 10.1016/j.survophthal.2005.06.010 16139038

[3] Dajee KP Abbey AM Williams GA. Management of dislocated intraocular lenses in eyes with insufficient capsular support. Curr Opin Ophthalmol (2016) 27(3):191–5. doi: 10.1097/ICU.0000000000000260 26913739

[4] Shen JF Deng S Hammersmith KM Kuo AN Li JY Weikert MP. Intraocular lens implantation in the absence of zonular support: an outcomes and safety update: A report by the american academy of ophthalmology. Ophthalmology (2020) 127(9):1234–58. doi: 10.1016/j.ophtha.2020.03.005 32507620

[5] Donaldson KE Gorscak JJ Budenz DL Feuer WJ Benz MS Forster RK. Anterior chamber and sutured posterior chamber intraocular lenses in eyes with poor capsular support. J Cataract Refract Surg (2005) 31(5):903–9. doi: 10.1016/j.jcrs.2004.10.061 15975454

[6] Khan MA Rahimy E Gupta OP Hsu J. Combined 27-gauge pars plana vitrectomy and scleral fixation of an akreos AO60 intraocular lens using Gore-Tex suture. Retina (2016) 36(8):1602–4. doi: 10.1097/IAE.0000000000001147 27388733

[7] Khan MA Samara WA Gerstenblith AT Chiang A Mehta S Garg SJ. Combined pars plana vitrectomy and scleral fixation of an intraocular lens using gore-tex suture: One-year outcomes. Retina (2018) 38(7):1377–84. doi: 10.1097/IAE.0000000000001692 28492433

[8] Khan MA Gupta OP Smith RG Ayres BD Raber IM Bailey RS. Scleral fixation of intraocular lenses using Gore-Tex suture: clinical outcomes and safety profile. Br J Ophthalmol (2016) 100(5):638–43. doi: 10.1136/bjophthalmol-2015-306839 26319945

[9] Botsford B Williams AM Conner IP Eller AW Martel JN. Scleral fixation of posterior chamber intraocular lenses using Gore-Tex suture: clinical outcomes from a single institution. J Vitreoretin Dis (2018) 2:276e281. doi: 10.1177/2474126418791701

[10] Wagoner MD Cox TA Ariyasu RG Jacobs DS Karp CL. Intraocular lens implantation in the absence of capsular support: A report by the American Academy of Ophthalmology. Ophthalmology (2003) 110(4):840–59. doi: 10.1016/S0161-6420(02)02000-6 12689913

[11] Su D Stephens JD Obeid A Borkar D Storey PP Khan MA. Refractive outcomes after pars plana vitrectomy and scleral fixated intraocular lens with gore-tex suture. Ophthalmol Retina (2019) 3(7):548–52. doi: 10.1016/j.oret.2019.02.012 31277795

[12] Huang CW Tsai CY Lai TT. Short-term outcomes of a modified technique for small-incision scleral-fixated intraocular lens implantation using Gore-Tex sutures. Graefes Arch Clin Exp Ophthalmol (2021) 259(7):1889–96. doi: 10.1007/s00417-021-05201-4 33914157

[13] Vote BJ Tranos P Bunce C Charteris DG Da Cruz L. Long-term outcome of combined pars plana vitrectomy and scleral fixated sutured posterior chamber intraocular lens implantation. Am J Ophthalmol (2006) 141(2):308–12. doi: 10.1016/j.ajo.2005.09.012 16458685

[14] Day HR Jr Durrani AK Kim SJ Patel S. Outcomes and complications of concurrent pars plana vitrectomy and scleral-fixated intraocular lens placement using gore-tex suture. J Vitreoretin Dis (2019) 4(2):119–24. doi: 10.1177/2474126419895691 PMC9976259 37008382

[15] Ma IH Tsai CY Yang CM Lai TT. Modified cow-hitch suture for repositioning of subluxated scleral-fixated rigid intraocular lens. Ophthalmic Surg Lasers Imaging Retina (2019) 50(3):179–82. doi: 10.3928/23258160-20190301-08 30893452

[16] Sakamoto T Nagase Y Takiguchi M Umehara N. A successful case of external stenting for bronchomalacia lasting over 20 years. Ann Thorac Surg (2019) 108(2):e103–4. doi: 10.1016/j.athoracsur.2018.12.055 30716290

[17] Raina UK Kumar B Bhattacharya S Saini V Gupta SK Goyal J. A comparison of sutureless flanged fixation and 4-point Gore-Tex fixation for scleral-fixated intraocular lenses: a pilot study. Digit J Ophthalmol (2022) 28(3):51–7. doi: 10.5693/djo.01.2022.08.001 PMC9635760 36405443

[18] Canabrava S Carvalho MS. Double-flanged polypropylene technique: five-year results. J Cataract Refract Surg (2023) 49(6):565–70. doi: 10.1097/j.jcrs.0000000000001154 36745851

[19] McAllister AS Hirst LW. Visual outcomes and complications of scleral-fixated posterior chamber intraocular lenses. J Cataract Refract Surg (2011) 37:1263–9. doi: 10.1016/j.jcrs.2011.02.023 21700103

[20] Asadi R Kheirkhah A. Long-term results of scleral fixation of posterior chamber intraocular lenses in children. Ophthalmology (2008) 115:67–72. doi: 10.1016/j.ophtha.2007.02.018 17481735

[21] Wilgucki JD Wheatley HM Feiner L Ferrone MV Prenner JL. One-year outcomes of eyes treated with a sutureless scleral fixation technique for intraocular lens placement or rescue. Retina (2015) 35:1036–40. doi: 10.1097/IAE.0000000000000431 25549073

[22] McKee Y Price FW Feng MT Price MO. Implementation of the posterior chamber intraocular lens intrascleral haptic fixation technique (glued intraocular lens) in a United States practice: Outcomes and insights. J Cataract Refract Surg (2014) 40:2099–105. doi: 10.1016/j.jcrs.2014.04.027 25457381

[23] Ohr MP Wisely CE. Refractive outcomes and accuracy of IOL power calculation with the SRK/T formula for sutured, scleral-fixated Akreos AO60 intraocular lenses. Graefe’s Arch Clin Exp Ophthalmol (2020) 258(10):2125–9. doi: 10.1007/s00417-020-04721-9 32504099

[24] Goel N. Clinical outcomes of combined pars plana vitrectomy and trans-scleral 4-point suture fixation of a foldable intraocular lens. Eye (2018) 32(6):1055–61. doi: 10.1038/s41433-018-0018-2 PMC5997732 29398696

